# Hypolipidemia contributing to the severity of sepsis triggered by influenza a virus: A case report

**Published:** 2020-12-11

**Authors:** Abdallah Qasim, Omar Kousa, Venkata Giri Andukuri

**Affiliations:** Department of Internal Medicine, Creighton University, Nebraska, United States

**Keywords:** hypolipidemia, severe sepsis, multiorgan failure, immune system activation, case report

## Abstract

Hypolipidemia, an increasingly diagnosed disorder, is defined as a low-density lipoprotein serum level of <50 mg/dL. Hypolipidemia can be asymptomatic. However, the effect of hypolipidemia on sepsis survival and severity is still to be identified. Multiple studies show the physiologic effects of cholesterol on the immune system, and other studies linked hypolipidemia to increased mortality and morbidity. In this case, we present a young patient admitted for severe sepsis, and he developed multiorgan failure. Workup revealed hypolipidemia. The patient recovered from sepsis with residual renal and cardiac injury. We hypothesized that hypolipidemia could be contributing to the increased morbidity in the patient, although further studies are needed to approve this hypothesis. What is unique about this case is that it sheds light on a commonly overlooked metabolic abnormality that plays a role in the body’s response to infections and sepsis.

**Relevance for patients:**

This case report presents a previously healthy young patient admitted for pneumonia who had a complicated course. Workup revealed hypolipidemia that can be contributing to the severity of his disease. This observation may lead to more studies to evaluate the relationship between lipoprotein level and disease severity which may change the management for patients with hypolipidemia, especially with the familial type.

## 1. Introduction

Lipid metabolism is an essential process in almost all types of cells. Disorders of lipid metabolism affect many organs such as cardiovascular diseases in the case of hyperlipidemia. Increased screening for lipid metabolism disorders has led to an increased diagnosis of hypolipidemia. Hypolipidemia affects different body systems, including the immune system, as it plays a role in activating and controlling the immune system. Hypolipidemia may play a role in decreased activation of the immune system and thus increased morbidity in sepsis patients. In this case, we emphasized the effect of hypolipidemia of sepsis morbidity. We present the following case in accordance with the CARE Guideline.

## 2. Case Presentation

A 46-year-old male presented in 2019 to the emergency department because of a 1-week history of dry cough, chills, and not feeling well. A review of systems was negative for exertional chest pain, shortness of breath, orthopnea, palpitation, constipation, weight changes, or recent skin rash. No recent travel history or sick contact. He has a past medical history of hypertension, not on medications. Social history was negative for smoking, alcohol use, or illicit drug use. Family history was significant for end-stage renal disease. He had no known allergies.

**Figure 1:**
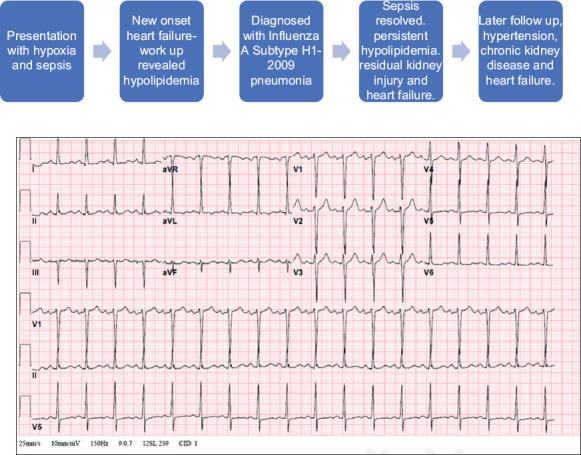
Electrocardiogram showed sinus tachycardia, voltage criteria for left ventricular hypertrophy, and nonspecific T wave abnormality.

**Figure 2:**
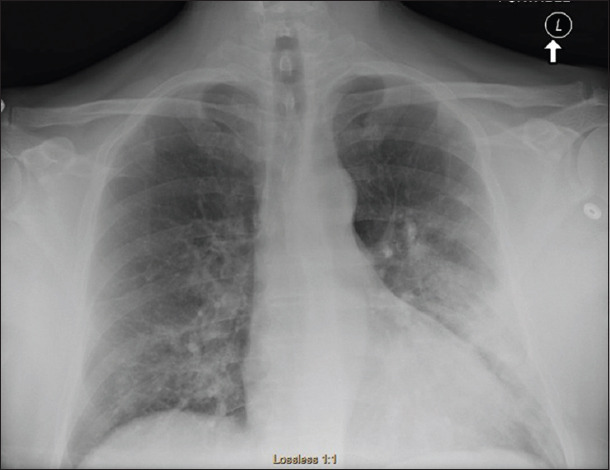
Chest radiograph showed that two frontal views of the chest are without comparison. Cardiac silhouette and pulmonary vasculature are normal. There is a left upper lobe infiltrate consistent with pneumonia. Epigastrium and skeletal structures are unremarkable.

On physical examination, he was found to be afebrile and hypoxic and required 3.5 L of oxygen to maintain oxygen saturation 91%. Blood pressure and heart rates were 160/108 mmHg and 108 beats/min, respectively. The patient’s weight was 110 kg and his height was 188 cm. Further examination revealed jugular venous distention, positive hepatojugular reflux, bilateral inspiratory crackles on lung auscultation, and minimal bilateral pitting edema. The rest of the examination was unremarkable. Initial laboratory workup revealed leukopenia, thrombocytopenia, elevated troponin, and creatinine (Table 1). Electrocardiogram showed sinus tachycardia with no significant ST changes (Figure 1). Chest radiograph showed left lower lobe consolidation suggestive of community-acquired pneumonia (Figure 2). The patient was resuscitated with three liters of lactate ringer, and blood culture and an influenza swab were taken. He was started on ceftriaxone and azithromycin and admitted for further management.

**Table 1:** Laboratory work up on presentation.

Test	Day-1	Day-3	Day-5 (On-discharge)	One month after discharge	Reference range
Hematology tests					
White blood cell (k/µL)	3.0	3.0	3.0	11.0	4-12 k/µL
Hemoglobin (g/dL)	13.8	13.2	12.7	14.7	12-16 g/dL
Platelet (k/µL)	124	185	219	302	140-440 k/µL
Neutrophils count (k/µL)	1.9	1.8	1.8	5.0	1.5-8.0 k/µL
Lymphocyte count (k/µL)	0.9	0.8	0.8	3.0	1.0-4.5 k/µL
Chemistry tests					
Glucose (mg/dL)	117	130	125	141	74-106 mg/dL
Sodium (mmol/L)	144	144	142	3.4	137-145 mmol/L
				141	
Potassium (mmol/L)	3.3	3.3	3.6	111	3.5-5.1 mmol/L
				3.4	
Chloride (mmol/L)	114	114	111	21	98-107 mmol/L
				111	
Carbon dioxide (mmol/L)	18	18	27	17	22-30 mmol/L
				21	
Urea (mg/dL)	36	36	21	1.5	9-20 mg/dL
				17	
Creatinine (mg/dL)	3.96	3.96	2.1	4.5	0.7-1.2 mg/dL
				1.5	
Lipase (u/L)	753	-	-	-	73-393 u/L
Total protein (g/dL)	7.2	5.8	6.1		6.0-8.4 g/dL
Albumin (g/dL)	3	3	3	4.5	3.5-5.0 g/dL
Calcium (mg/dL)	7.9	-	-	9.4	8.4-10.2 mg/dL
Magnesium (mg/dL)	1.7	1.5	1.5	2.5	1.8-2.6 mg/dL
Phosphorus (mg/dL)	3.1	2.7	3.1	3.5	2.5-4.9 mg/dL
Total bilirubin (mg/dL)	0.9	0.7	0.9	1.2	0.2-1.3 mg/dL
Aspartate aminotransferase (IU/L)	165	134	76	27	15-46 IU/L
Alanine transaminase (IU/L)	76	60	44	30	4-50 IU/L
Alkaline phosphates (IU/L)	92	86	103	-	38-126 IU/L
Creatine kinase (IU/L)	3395	3575	1216	-	55-170 IU/L
Brain natriuretic peptide (pg/ml)	1057	-	-	-	<449 pg/ml
Thyroid-stimulating hormone (UIU/L)	0.593	-	-	-	0.4-3.8 UIU/L
Free T4 (ng/dL)	0.593	-	-	-	0.7-1.4 ng/dL
Troponin (ng/mL)	0.28	-	-	-	<0.04
Procalcitonin (ng/mL)	1.14	-	-	-	<0.05 ng/mL
C-reactive-protein (mg/L)	7.5	-	-	-	<9.0 mg/L
Coagulation studies					
Prothrombin time (s)	10.9	-	-	-	9.6-12 s
International randomized ratio	1	-	-	-	0.9-1.1
Urine Labs					
Urine blood	Positive	-	-	-	Negative
Urine RBC	3-5	-	-	-	3-5 cells
Urine protein	100	-	-	30	0 mg/dL
Labs for secondary hypertension					
Aldosterone (ng/dL)				10.4	0.0-30 ng/dL
Renin				<0.167	0.167-5.38
Parathyroid hormone (pg/mL)				186	18.4-80.0 pg/mL
Metanephrine (nmol/L)				**0.93**	**0-0.89 nmol/L**
Normetanephrine (nmol/L)				0.22	0-0.49 nmol/L

Further cardiac evaluation revealed stable troponin at 0.27 ng/dL, a transthoracic echocardiogram showed an ejection fraction of 30-35%, eccentric left ventricular hypertrophy, and global hypokinesia, his lipid profile was significant for low low-density lipoprotein (LDL) (Table 2), hemoglobin A1-C of 5.5, and apolipoprotein ration of 0.6, which means low risk for ischemic heart disease. His acute kidney injury was determined to be secondary to pre-renal etiology as renal ultrasound showed the normal sonographic appearance of the kidneys and urinary bladder, with no signs of hydronephrosis or obstructive uropathy.

**Table 2:** Laboratory lipid and cardiac workup.

	Day-1	Day-3	Reference range
Cholesterol (mg/dL)	52	75	120-200 mg/dL
Triglyceride (mg/dL)	153	231	<149 mg/dL
High-density lipoprotein (mg/dL)	26	28	40-60 mg/dL
Low-density lipoprotein (mg/dL)	<5	<5	<99 mg/dL
Hemoglobin-A1C (%)	5.5	-	4.5-5.6%
Apolipoprotein B/A ration	0.6	-	(0.3-0.4) one half risk for coronary artery disease

**Figure 3:**
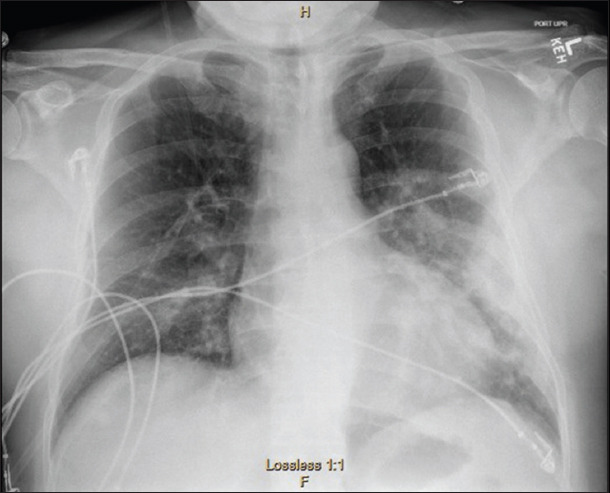
Chest radiograph showed unchanged airspace opacities within the left midlung zone. No pleural effusion or pneumothorax. Cardiomediastinal silhouette within normal limits. Pulmonary vasculature within normal limits. Unchanged osseous structures.

His overall clinical picture was secondary to severe underlying sepsis, which resulted in acute myocardial and kidney injury. Overnight, he continued to required oxygen supplementation to correct his ongoing hypoxemia, for which he had a chest X-ray to rule out for fluid overload component (Figure 3), which showed unchanged left lower lobe opacity. On day 2 of admission, his blood culture remained negative for growth, and rapid influenza screen was negative for A and B serotype. Respiratory pathogen panel came back later with influenza A subtype H1-2009, and he was subsequently started on an adjusted renal dose of oseltamivir. On day 5, he was off oxygen supplementation, and his labs improved dramatically (Table 1) and he was subsequently discharged on oseltamivir, doxycycline, hydralazine, and carvedilol on a stable condition with close follow-up with his primary care physician and nephrology. His feeding was enteral (a low-salt diet) all through his hospitalization.

One month later, he showed at the nephrology clinic, his blood pressure and heart rates were 214/136 mmHg and 112 beats/min, respectively. Further workup was sent for his underlying chronic kidney disease and secondary hypertension etiologies, with as-needed clonidine for better blood pressure control. Results showed normal metanephrine levels and mildly elevated normetanephrine levels but did not reach the threshold for pheochromocytoma (Table 1). Serum aldosterone to renin ratio is abnormal at 34, which was determined that his hormonal imbalance could be contributing to uncontrolled blood pressure (Table 1). He was subsequently started on spironolactone 25 mg once a day. On a 2-week phone appointment follow-up, he reported blood pressure readings between 130/150 over 80-85 mmHg. He was advised to follow within a month for repeated blood workups and further imaging studies for suspected Conn syndrome.

Case timeline as the following:

## 3. Discussion

Cholesterol level is frequently tested in many patients for the screening of dyslipidemia, and some of them have abnormally low cholesterol levels. Hypolipidemia, defined as of LDL level <50 mg/dL, is described by absent or decreased concentration of apolipoprotein B-containing lipoproteins. Hypolipidemia is either acquired or inherited.

Familial hypobetalipoproteinemia is an inherited autosomal codominant disorder. In the homozygous form, the patients usually have very low or absent LDL cholesterol, and they are usually symptomatic in their childhood, similar to another syndrome called abetalipoproteinemia, which is autosomal recessive. Heterozygous familial hypobetalipoproteinemia patients are usually asymptomatic and have decreased LDL cholesterol [1,2]. Hypolipidemia can also be acquired by multiple diseases, which include cancers (colorectal, prostatic carcinoma, leukemias, myeloma, and other monoclonal gammopathies), malabsorption, anemia, and severe illness [3].

Lipids are essential in many organs and physiological processes because of which the patients who have abetalipoproteinemia – the patients who are unable to synthesize LDL cholesterol – present with multisystemic signs and symptoms affecting vision, liver, peripheral nerves, and absorption of the gastrointestinal tract [4]. The role of cholesterol in immunity is getting increasingly recognized with multiple observational studies that linked low level of LDL with higher mortality and poor prognosis in the patient having severe infections such as severe sepsis or severe pneumonia [5,6], and a low cholesterol level in sepsis patients has been suggested as a marker for a worse prognosis. Multiple studies link heterozygous hypolipidemia to a decreased inflammatory response to infection and higher risk for severe infections and sepsis.

Studies have shown that LDL cholesterol has a pro-inflammatory effect, which helps the body to augment the inflammatory response in cases of infection or injury. On the other hand, high-density lipoprotein cholesterol helps to decrease inflammation, which shows that cholesterol has a pro-inflammatory and anti-inflammatory effect, and a balance between them helps to control the inflammatory process. By this means, having very low LDL cholesterol will adversely affect the ability of the body to augment the inflammatory process, which will contribute to higher morbidity in sepsis patients, such as what happened in our patient [7-9].

As mentioned earlier, hypolipidemia can be acquired or congenital. One of the reasons for acquired hypolipidemia is stress, such as severe sepsis. However, in our patient, it is still unknown which came first, is it the severe sepsis that caused acquired hypolipidemia and stress cardiomyopathy or that the patient is more prone to severe sepsis because he has hypolipidemia. He had more morbidity and multiorgan failure. However, in such a young patient without comorbidities, having such severe sepsis is somewhat unusual, and this makes us think that the patient is maybe already prone to severe infection because of the hypolipidemia causing the decreased response to infections and what supports this is that the lipid profile repeated after recovering from the acute sepsis and it still showed low LDL.

Although multiple studies link severe infections to low cholesterol levels by decreased production due to changes in genetic expression induced by severe sepsis, in these cases, the cholesterol levels normalized after the resolution of the acute sepsis, which did not happen with our patient [10]. Walley *et al*. did a retrospective analysis on patients admitted with sepsis who has mutations in proprotein convertase subtilisin/Kexin type 9 and 3-Hydroxy-3-Methylglutaryl-CoA reductase enzymes, which are associated with low LDL levels. The analysis did not show a causal relationship of low level of LDL with increased mortality in sepsis patients. However, the study was done on the genetic mutations that are associated with low LDL and not actual LDL level. Moreover, our case suggests a possible association of low LDL with increased morbidity in sepsis triggered by influenza A pneumonia [11].

This case report shines a light on one of the factors that might affect sepsis patients’ morbidity and survival and affects prognosis. This study is limited by being a case report of a single case. The conclusion should be further investigated by more extensive studies.

## 4. Conclusion

Severely low LDL levels can be associated with higher morbidity in severe sepsis patients. In addition, there might be a possible association of severely low LDL levels with congestive heart failure. However, further clinical studies are needed to evaluate the effect of severely low cholesterol levels on the inflammatory response, especially with sepsis patients. And whether changing the diet to address the deficiency would help with that or not is a question that is still needs to be answered by further studies.
